# Associations between urine glyphosate levels and metabolic health risks: insights from a large cross-sectional population-based study

**DOI:** 10.1186/s12940-024-01098-8

**Published:** 2024-06-27

**Authors:** Sarah Otaru, Laura E. Jones, David O. Carpenter

**Affiliations:** 1 Department of Environmental Health Sciences, University at Albany, State University of New York, 1 University Place, Rensselaer, NY USA; 2 Institute for Health and the Environment (IHE), 5 University Place, Rensselaer, NY USA; 3 Center for Biostatistics, Bassett Research Institute, 1 Atwell Rd., Cooperstown, NY USA; 4 Department of Epidemiology and Biostatistics, School of Public Health, State University of New York, 1 University Place, Rensselaer, NY USA

**Keywords:** Exploratory factor analysis, Quantitative score, Metabolic syndrome, MetS, NHANES, Albuminuria, Glyphosate

## Abstract

**Background:**

The prevalence of metabolic syndrome (MetS) in American adults increased from 37.6% in the 2011–12 period to 41.8% in 2017–2018. Environmental exposure, particularly to common compounds such as glyphosate, has drawn increasing attention as a potential risk factor.

**Methods:**

We employed three cycles of data (2013–2018) from the National Health and Nutrition Examination Survey (NHANES) in a cross-sectional study to examine potential associations between urine glyphosate measurements and MetS incidence. We first created a MetS score using exploratory factor analysis (EFA) of the International Diabetes Federation (IDF) criteria for MetS, with data drawn from the 2013–2018 NHANES cycles, and validated this score independently on an additional associated metric, the albumin-to-creatinine (ACR) ratio. The score was validated via a machine learning approach in predicting the ACR score via binary classification and then used in multivariable regression to test the association between quartile-categorized glyphosate exposure and the MetS score.

**Results:**

In adjusted multivariable regressions, regressions between quartile-categorized glyphosate exposure and MetS score showed a significant inverted U-shaped or saturating dose‒response profile, often with the largest effect for exposures in quartile 3. Exploration of potential effect modification by sex, race, and age category revealed significant differences by race and age, with older people (aged > 65 years) and non-Hispanic African American participants showing larger effect sizes for all exposure quartiles.

**Conclusions:**

We found that urinary glyphosate concentration is significantly associated with a statistical score designed to predict MetS status and that dose–response coefficient is nonlinear, with advanced age and non-Hispanic African American, Mexican American and other Hispanic participants exhibiting greater effect sizes.

**Supplementary Information:**

The online version contains supplementary material available at 10.1186/s12940-024-01098-8.

## Background

Metabolic syndrome (MetS) consists of a constellation of conditions—high blood pressure, high blood sugar levels, excess abdominal fat, and elevated cholesterol and triglyceride levels—that collectively amplify the risk of cardiovascular disease, stroke, and type 2 diabetes mellitus [3]. In the United States, prevalence of MetS increased from 37.6% in the 2011–12 period to 41.8% in 2017–2018 among adults, as shown by an analysis of data from the National Health and Nutrition Examination Survey (NHANES) [34]. This upward trend underscores the urgency of understanding and mitigating the factors contributing to MetS.

Risk factors for MetS are broadly categorized into nonmodifiable and modifiable factors. Age, sex, and race/ethnicity are nonmodifiable factors that can influence the likelihood of developing MetS, with variations observed across different demographic groups. Diet, physical activity, weight, and environmental chemical exposure are modifiable factors. Among these, the role of environmental chemical exposure, particularly to common compounds such as glyphosate, has drawn increasing attention.

Glyphosate is the most widely used broadleaf herbicide globally and is marketed as Roundup™ in the United States [8]. It blocks a pathway required for synthesis of aromatic amino acids found in plants but not animals [49], although some gut bacteria are sensitive, and is applied to eliminate weeds and grasses. Since its introduction in the 1970s, usage has increased by a factor of at least 100 due in part to introduction of glyphosate tolerant “Roundup™-ready” cereal crops [8]. A major use of glyphosate is to kill weeds before harvesting grain crops, and it is also applied directly to grain and pulse crops to speed desiccation [45]. These practices, however, lead to glyphosate residues in foods made of wheat, oats and other grains, as well as beans and lentils, contributing to exposure via dietary intake among the general population [45]. In addition to dietary exposure, inhalation and dermal contact are significant exposure pathways for farmers and others who apply Roundup™ [12].

Although glyphosate was originally thought to have no effect on humans, it is now rated by the International Agency for Research on Cancer (IARC) as a probable human carcinogen [21], and has effects on both female and male fertility [54],has neurological effects [1, 36] and causes mitochondrial damage [46]. Animal and in vitro studies indicate that glyphosate exposure interferes with glucose uptake into adipocytes [13, 47], is associated with liver fibrosis [40], increases apoptosis [11, 20], induces oxidative stress [38, 46], and alters the gut microbiome [24, 33, 39, 48, 51, 59]. These mechanisms are linked to the pathogenesis of MetS [9, 35].

Epidemiological research exploring the association between glyphosate and MetS is limited, with only two studies conducted thus far. The CHAMACOS Study by [14], focused on mother–child dyads and revealed a significant association between glyphosate exposure and MetS risk by young adulthood. Similarly [14, 17], observed a positive association between glyphosate levels and MetS among U.S. adults. However, both of these studies have limitations. Eskenazi et al. studied a small farming population with high levels of multiple chemical exposures, leading to poor generalizability to the U.S. population. Glover et al. [17] adjusted for features intrinsic to the syndrome itself, such as hypercholesterolemia, hypertension, and diabetes, potentially biasing estimations of the associations between glyphosate and metabolic syndrome. These studies also employ threshold-based definitions of MetS based on dichotomized continuous variables, leading to loss of information and potentially statistical power. Clinical studies suggest that it is desirable to include continuous variables to create accurate scoring systems for MetS [10, 26].

Given the limitations in the available studies and the problems associated with the use of threshold-based definitions from a research standpoint, our study aims are twofold: (1) to create and validate a score that captures the continuous nature of risk factors that comprise MetS and (2) to employ this score in linear regression analyses that explore associations between urine glyphosate concentrations and MetS. We anticipate that our approach will provide a more nuanced understanding of how glyphosate exposure affects the risk of MetS.

## Methods

### Study population

We employ data from the National Health and Nutrition Examination Survey (NHANES), accessible via the Centers for Disease Control and Prevention (CDC) website (https://www.cdc.gov/nchs/nhanes), in a cross-sectional study to examine potential associations between urine glyphosate measurements and metabolic syndrome (MetS). NHANES is a comprehensive research initiative designed to assess the health and nutritional status of adults and children in the United States, surveying approximately 5,000 individuals annually through interviews, physical examinations, and laboratory investigations. The research protocols were sanctioned by the National Center for Health Statistics of the U.S. CDC, and informed consent was obtained from all participants.

**Fig. 1:**
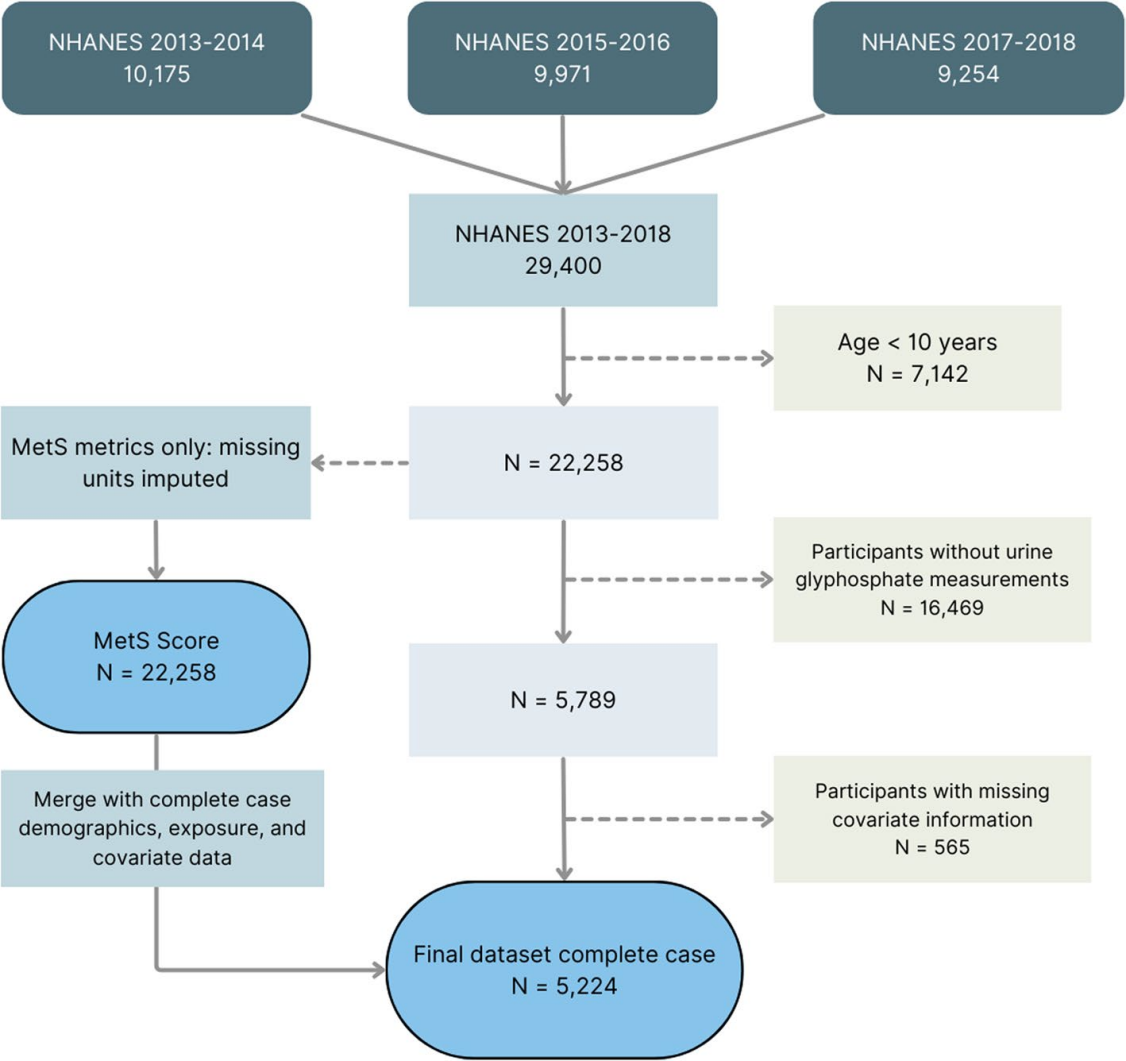
Workflow and sample sizes showing sample size evolution from the initial raw sample (three NHANES cycles, 2013–2018) to the final complete case sample with the created MetS score

Our analysis included NHANES datasets from 2013–2018 (3 cycles) with urinary glyphosate measurements. Glyphosate measurements were obtained from one-third of the participants who consented to future analysis of their laboratory samples. Therefore, from the initial pool of 29,400 participants, 22,333 were excluded due to a lack of urine glyphosate data and incomplete covariate information. Since the earliest symptoms of metabolic syndrome do not occur or are undefined among the very young [30], children under the age of 10 years (*n* = 1180) were excluded, yielding a sample size for the primary analysis of 5224 (Fig. 1).

### Exposure: urine glyphosate

Urinary glyphosate levels were measured in a 200-µl urine sample utilizing a 2D online ion chromatography system paired with tandem mass spectrometry (IC-MS/MS) along with isotope dilution for quantification. The results are expressed in nanograms per milliliter (ng/ml), with the assay sensitivity established at a lower limit of detection (LLOD) of 0.2 ng/ml. For our analysis, we categorized urinary glyphosate levels by quartile (see Table 1 for a summary of exposure and outcome).

**Table 1:** Baseline characteristics of the complete case study population (*N* = 5224). Sample size is limited by the number complete cases for glyphosate and creatinine measurements

**Covariate**	**Level**	**Male** (*n* = 2605)			**Female (***n* = 2619)	
Sex	Male	2605 (100.0)			-	
	Female	-			2619 (100.0)	
Age Category Ref: < 19 years	< 19yrs	619 (23.8)			537 (20.5)	
	20-39yrs	646 (24.8)			678 (25.9)	
	40-59yrs	659 (25.3)			716 (27.3)	
	> 60yrs	681 (26.1)			688 (26.3)	
Race-Ethnicity Ref: White	White (ref)	1021 (39.2)			958 (36.6)	
	Mexican American	412 (15.8)			421 (16.1)	
	Other Hispanic	244 (9.4)			279 (10.7)	
	Black	519 (19.9)			552 (21.1)	
	Asian	276 (10.6)			293 (11.2)	
	Multiracial	133 (5.1)			116 (4.4)	
BMI Mean (SD)		27.71 (6.83)			28.78 (8.07)	
Income-Poverty Ratio Med [IQR]		2.02 [1.06, 3.91]			1.94 [1.03, 3.82]	
		Mean (Median) values by Quartile (*N* = 5789)				
Urinary Metric	Min	Quartile 1	Quartile 2	Quartile 3	Quartile 4	Max
Creatinine^a^	0.035	0.40 (0.64)	0.89 (1.12)	1.39 (1.72)	2.39	6.0
Glyphosate^b^	0.071	0.12 (0.14)	0.26 (0.34)	0.45 (0.61)	1.22	8.2
ACR^c^	0.31	3.7 (4.9)	6.1 (7.5)	10.1 (14.3)	157	21152
MetS Score	-1.80	-0.89 (-0.56)	-0.31 (0.08)	0.17 (0.47)	1.2	4.2

^a^Units: mg/dL

^b^Units: ng/mL

^c^Units: mg/gm

### Outcome: metabolic syndrome score

Metabolic syndrome was defined roughly per the International Diabetes Federation (IDF) criteria and includes the presence of central obesity and any two of four additional risk factors [2, 4, 30]. We included a total of 5 features based on biometric measurements and laboratory assessments reported in the NHANES. Following work by Cavero et al. [10], we included HbA1c as a dysglycemia indicator [10], as well as the composite risk factors triglyceride-to-HDL ratio and mean arterial pressure (MAP) in our selected features, and extracted a single factor score as described below. The mean arterial pressure (MAP) was estimated as SBP + 1/3 (SBP—DBP). The selected risk and composite factors include the following:

- Waist circumference
- Fasting glucose
- glycoheme (HbA1c)
- Triglyceride-to-HDL ratio
- Mean arterial pressure (MAP)

These measures are common to most definitions of MetS [3, 6, 15, 19, 30, 61] for a comprehensive list of definitions with citations.

### Potential confounders/effect modifiers

Potential confounders in this study included participant age, sex, race/ethnicity, BMI, and family income-to-poverty ratio, which ranged from 0 to 5. Age was categorized into four groupings as follows: 10 to 19 years, 20 to 39 years, 40 to 59 years, and 60 years and above. The reference level was 10 to 19 years. Race was categorized as Non-Hispanic White (white), Non-Hispanic Black (Black), Non-Hispanic Asian (Asian), Mexican–American, Other Hispanic, or Non-Hispanic Multiracial (Multiracial), with a reference level of white. Finally, we included urine creatinine measurements as a covariate to adjust for variations in the concentration of urinary analytes due to dilution effects [7].

### Statistical analyses

#### Factor analysis to create a MetS index

We used exploratory factor analysis (EFA) to create a single index from the five risk factors described above (waist circumference, triglyceride-to-HDL ratio, fasting glucose, glycoheme (HbA1c), and MAP). EFA works by capturing common variance in one or more directions in the multivariable space of interest, among MetS risk factors. Each of these “directions” is a factor. After extracting significant factor(s) from our analysis, we obtained a table of weightings that may be used to create the index or indices. These indices can be considered weighted sums of the variables of interest. A goal of our study was to construct a working single-factor model for MetS, and our model is a modification of one of three models developed and tested by Cavero-Redondo et al. [10], including both HbAlc and fasting glucose.

As missing values for the MetS risk factors ranged from 7.9% to 61.5%, we first imputed the data using sorted and grouped hot-deck single imputation, which involves randomly selecting appropriate donors from the existing distributions in specified columns after sorting by age category and grouping by sex. Grouped and sorted hot-deck is an efficient donor-based method that works well for large datasets with mixed continuous and categorical variables, and for calculations that do not directly involve inference [37]. Data driven donor-based methods are conservative in that they draw samples from existing distributions. This approach yields a sample size of 22,258 on the five features selected for the EFA process. Following imputation, an average systolic blood pressure measurement was created by averaging the first three systolic blood pressure measurements. We then computed the MAP, estimated as SBP + 1/3 (SBP—DBP). Since HDL levels vary inversely with MetS incidence and we sought a positively weighted score, we computed the triglyceride-to-HDL ratio, a measure of cardiovascular risk found in all three candidate MetS score models tested by Cavero-Redondo et al. [10]. We then standardized the data, and since factor analysis is a linear method that does not respond well to highly correlated data, we checked for excessive multicollinearity by computing a correlogram and confirming that the selected metrics all had pairwise correlations less than 0.8 (see Supplemental Fig. 1 for the correlogram). Finally, the appropriateness of the EFA was checked by running the Kaiser–Meyer–Olkin (KMO) measure of sampling adequacy test [27, 29] and Bartlett’s sphericity test (*p* < 0.00001; [53]). The KMO test measures the proportion of common variance in a group of variables; the higher the statistic is (closer to one), the more appropriate the grouping is for EFA [28]. Our KMO values ranged from 0.65 to 0.72 (median 0.67), and our selected features were confirmed to be adequate for factor analysis. Bartlett’s sphericity test indicates that the correlation matrix of the selected features is suitable for detecting structure.

As EFA assumes multivariate normality for extracting factor loadings via maximum likelihood, MetS metrics were first visualized and then log-transformed since all data were slightly skewed [53]. Parallel analysis by Horn’s test of principal components or factors indicated that one factor was sufficient to explain common variance in the five risk features [23]; see Supplemental Fig. 2 for a Parallel Analysis Plot. EFA was performed on the standardized metrics, and the score for one factor was extracted. Factor loadings > 0.4 were considered criteria for inclusion in the MetS model, and all five selected features met this criterion for inclusion. The EFA procedure assumed orthogonality, was computed on a correlation matrix, and used a varimax rotation. As a limited check on the single imputation approach, we also multiply imputed the data, creating 10 imputed datasets via the grouped and sorted hot-deck method described above. After imputation, correlation matrices were calculated for each imputed dataset and pooled following a method outlined by Nassiri [42]. EFA was then performed on a single pooled correlation matrix and a score extracted as above. All imputation and EFA were performed in the R programming language (R 4.3.0) using the “hot-deck” algorithm from the VIM library [31], parallel analysis from the paran library, and “factanal” from the psych library.

#### Validation of the MetS score

While we lacked many IDF metrics for validating the MetS score, NHANES does provide ACR, the ratio of urine albumin to creatinine, available as the “URDACT” variable (see Table 1 for a summary of ACR by quartile). Neither creatinine nor albumin measurements are used in the creation of the score, so this provides at least one opportunity to test the proposed MetS score for MetS-associated symptoms; however, of the three test metrics, the ACR score was the least correlated with the MetS scores developed by Cavero-Redondo et al. [10]. An ACR score above 30 and below 300 indicates “microalbuminuria,” but this range of transitional effects comprises an order of magnitude. Microalbuminuria is an indicator of kidney disease, is associated with diabetes and was recently included in the comprehensive definition of MetS proposed by the World Health Organization [52]. However, while microalbuminuria is more common in MetS patients, it is not unique to MetS. We validated the MetS score proposed in this paper using a logistic model as a classifier and a dataset that included only the MetS and ACR scores (*n* = 20,765). Cutoff values of ACR > 30 (microalbuminuria), ACR > 100 (microalbuminuria) and ACR > 300 (macroalbuminuria) were selected, and binary ACR variables were created based on these values. The data were divided into training and testing sets using a stratified sampling scheme to ensure that nonzero ACS indicator values appeared in both the testing and training sets. Logistic models were fit on the training set, and predictions were made on the test set, with the model error rate, sensitivity, specificity, accuracy and diagnostic odds ratio [16] computed.

### Bivariate and multivariate analysis

Bivariate analysis was conducted using linear regressions, with t tests or ANOVA (analysis of variance) for continuous variables to examine the exposure and outcome across categorical covariate levels.

We employed multivariable linear regression models to explore adjusted associations between urinary glyphosate levels and the MetS index. Confounders were selected using a directed acyclic graph (DAG) and included age category, race, sex, BMI, and income-poverty ratio (see Supplemental Fig. 4). Creatinine was included as a linear term to adjust for urine dilution [7]. BMI and creatinine levels were continuous, though creatinine was square-root transformed and both covariates were then standardized before regressions were run. The MetS score was standardized to facilitate comparisons across stratified models, and the exposure was categorized by quartile to allow for potential nonlinearities. Finally, we assessed for effect modification by age category, race and sex [55], stratifying on each variable and repeating the analysis on each stratum. Since this is an exploratory study, we do not adjust for multiplicity. All statistical analyses were performed using the R programming language (R version 4.3.3 and a *p*-value of less than 0.05 indicated statistical significance.

## Results

Baseline characteristics of the study population (*n* = 5,224) are summarized in Table 1, stratified by sex. The characteristics of all three NHANES cycles utilized, including missing units, are summarized in Supplemental Table 1, and the workflow for data cleaning and sample assembly is shown in Fig. 1. From the initial sample, we first omitted children under the age of 10 (*n* = 7,142), leaving a sample size of 22,258. We then omitted participants lacking information on urinary glyphosate (74% missing), creatinine (6.7% missing), BMI (5.5% missing) or poverty-income ratio information (10.7% missing). The degree of missing units in the exposure makes imputation of the exposure for inference purposes impossible. However, a comparison of summary information from the complete case sample (Table 1) and the original dataset (Supplemental Table 1) suggests the complete case sample is representative. The sample is roughly balanced by sex, with 2,605 males and 2,619 females. The largest single ethnic group is Non-Hispanic White (about 38%), followed by Non-Hispanic Black (about 20%). Hispanics as a group (Mexican American and ‘Other Hispanic’) comprised about 25%. Non-Hispanic Asians comprised about 22% and multiracial participants less than 10%. The cohort was older, with 52% of participants age 40 or over, and 26% age 60 or over. Variables used in EFA (*n* = 22,258) to create the MetS score were imputed using a conservative donor-based single-imputation method before proceeding with EFA (see Fig. 1 for details). Features used in the EFA to create the MetS score are summarized in Table 2.

**Table 2:** Variables used to create the metabolic syndrome (MetS) score (*n* = 22,258)

Feature^a^	Min	25%	50%	75%	Max
**Waist circumference**	49.5	81.8	94.5	107	177.9
Systolic blood pressure	64.67	108	117.33	130.67	231.33
Diastolic blood pressure	0	60	68	76	135.33
**Glycoheme (HbA1c)**	3.5	5.2	5.5	5.8	17.5
**Fasting Glucose**	21	93	100	109	479
HDL	6	42	51	62	226
Triglycerides	10	57	85	129	4233
**Triglyceride: HDL Ratio**	0.13	1.03	1.65	2.69	103.24
**Mean Arterial Pressure (MAP)**	71.56	122.89	134.44	150.22	279.56

^a^Variables and composite variables (the latter shown below the double line at the bottom of the table) used in the score are in boldface. MAP = SBP + 1/3 (SBP—DBP). The MetS score is estimated on as many samples as possible to create a score broadly applicable to the general population. The missing data are summarized in Supplemental Table 2

**Fig. 2:**
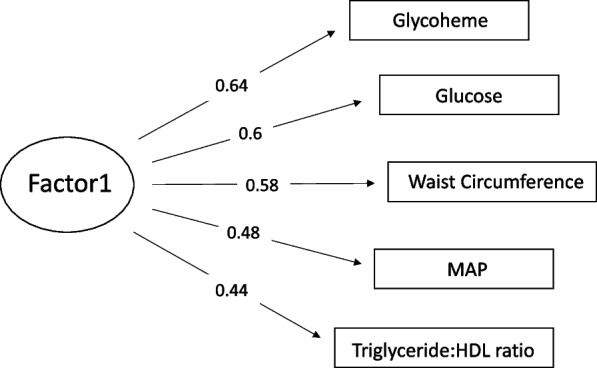
Factor analysis diagram. Variable loadings for the MetS index were created via factor analysis on hot-deck imputed risk factor metrics (Table 2)

Exploratory factor analysis resulted in positive weightings and yielded a single index score. The weightings are shown in a factor analysis diagram (Fig. 2): glycoheme and glucose dominate, but just slightly. Biometric measurements including waist circumference and MAP were similarly weighted and triglyceride:HDL ratio weighted the least, though well within the range of inclusion. Weightings were identical for the score obtained from EFA on multiply imputed risk factor metrics. Summary statistics for the indices are listed by quartile in Table 1 and for the component variables, Table 2. Distributions and missing units for the component variables are summarized in Supplemental Table 2.

As demonstrated in Cavero-Redondo et al. [10], we validated our score by using it to predict vascular damage (associated with MetS) according to the albumin-to-creatinine ratio (e.g., ACR $\ge 30 mg/g$), a typical, though not alone sufficient, metric of clinical performance for a MetS score [52, 56, 57]. Results from the validation procedure suggested that the sensitivity, specificity and accuracy increase with the ACS cutoff (Supplemental Table 3), with an error rate of 12% for an ACR cutoff of 30 and above (microalbuminuria) and 2% for an ACR cutoff of 300 and above (albuminuria). Again, because microalbuminuria is associated with MetS but is not completely predictive of it, this is not an ideal test.

We explored bivariate associations between the outcome and exposure plus patterns of bivariate associations between both the outcome and the exposure with covariates identified as confounders or potential effect modifiers (age, sex, race). Exposure and outcome are summarized by quartile in Table 3A-C. Unadjusted associations between the standardized MetS outcome score and glyphosate concentration, categorized by quartile, were significant for the reference quartile and quartiles 3 (Q3) and 4 (Q4), suggesting an inverted U-shaped dose‒response (maximum at Q3; Table 3A).

**Table 3:** Bivariate analysis. Note that every level value is relative to reference

**A.** Bivariate association between MetS score outcome and glyphosate level by exposure quartile				
**Glyphosate**	**Estimate**	**95% CI**	***p* value**	
Quartile 1 (ref)	-0.072	-0.126, -0.018	-	
Quartile 2	0.071	-0.005, 0.148	0.069	
Quartile 3	0.127	0.051, 0.204	0.001	
Quartile 4	0.090	0.014, 0.167	0.021	
**B.** Exposure and outcome by race/ethnicity				
**Log(Glyphosate) exposure by Race**				
**Race/Ethnicity**	**Estimate**	**95% CI**	***p* value**	**Pr(> F)**
White (ref)	0.530	0.504, 0.555	-	F = 8.35 *p* < 0.0001
Mexican–American	-0.076	-0.123, -0.029	0.002	
Other Hispanic	-0.030	-0.086, 0.026	0.291	
Black	0.038	-0.005, 0.081	*0.083*	
Asian	-0.110	-0.164, -0.055	0.0001	
Multiracial	0.037	-0.039, 0.114	0.33	
**Standardized MetS score (outcome) by Race**				
White (ref)	-0.040	-0.084, 0.004	*-*	F = 19.0 *p* < 0.0001
Mexican–American	0.089	0.008, 0.170	0.030	
Other Hispanic	0.083	-0.013, 0.180	*0.090*	
Black	0.112	0.038, 0.187	0.003	
Asian	-0.063	-0.156, 0.031	0.188	
Multiracial	0.018	-0.114, 0.149	0.792	
**C.** Exposure and Outcome by Age Category				
**Log(Glyphosate) exposure by Age Grouping**				
**Age Category**	**Estimate**	**95% CI**	***p* value**	**Pr(> F)**
10-19yrs (ref)	0.586	0.553, 0.62	-	F = 22.6 *p* < 0.0001
20-39yrs	-0.136	-0.182, -0.09	< 0.0001	
40-59yrs	-0.125	-0.170, -0.08	< 0.0001	
> 60yrs	-0.025	-0.070, 0.02	0.281	
**Standardized MetS score (outcome) by Age Grouping**				
10-19yrs (ref)	-0.695	-0.745, -0.645	-	F = 4113 *p* < 0.0001
20-39yrs	0.385	0.317, 0.454	< 0.0001	
40-59yrs	0.966	0.898, 1.034	< 0.0001	
> 60yrs	1.308	1.240, 1.376	< 0.0001	
**D.** Exposure and outcome by sex				
**Glyphosate exposure by Sex**				
**Sex**	**Estimate**	**95% CI**		***p* value**
Male (ref)	0.546	0.524, 0.568		-
Female	-0.067	-0.099, -0.036		< 0.0001
**Standardized MetS score (outcome) by Sex**				
Male (ref)	0.070	0.031, 0.108		-
Female	-0.139	-0.193, -0.085		< 0.0001

We found two race-ethnicity levels (Mexican–American and NH Asian) with significantly different mean estimates from the reference (White) for log-transformed glyphosate from regression (Table 3A; *p* < 0.0001). For the outcome MetS score, Mexican–American and Black participants had significantly higher scores than the reference group, and the ANOVA results were also highly significant (*p* < 0.0001) (Table 3B). A visualization of differences in exposure by race is shown in Supplemental Fig. 3A.

Unadjusted regressions of exposure and outcome with age category are consistent, showing significant differences in exposure between the reference level (ages 10–19) and ages 20–39 and 40–59 years, which are slightly reduced relative to the reference level (*p* = 0.004). Interestingly, the estimates for patients ages 60 years and older were not significantly different than the reference level (Supplemental Fig. 3B). The MetS score was significantly different across all age categories, which was unsurprising (Table 3C). Finally, there were significant differences in exposure and outcome levels according to sex (reference Male); women had, on average, slightly but significantly lower exposure levels (*p* < 0.0001) and slightly lower MetS scores than men did (*p* < 0.0001), from unadjusted regressions (Table 3D). As expected, the results for the MetS score did not significantly differ across races/ethnicities; however, the score increased with age and was slightly greater among men than women.

**Fig. 3:**
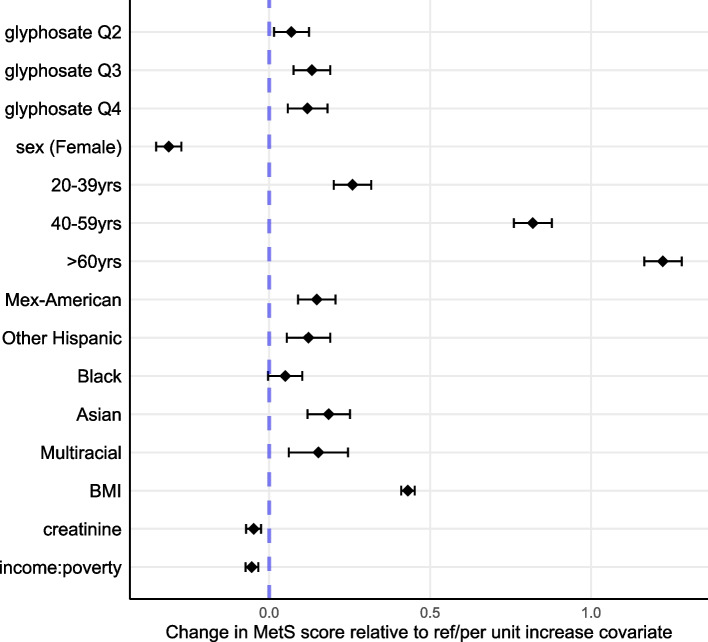
Change in MetS score by Quartile Glyphosate, full model with all covariates (*n* = 5,224). Model was adjusted as shown for sex (reference Male), age category (reference 10–19 years), race-ethnicity (reference Non-Hispanic White), scaled BMI, standardized creatinine, and standardized income-poverty ratio. See Table 4 for estimates, confidence intervals, and *p*-values

Adjusted associations between the standardized metabolic score and glyphosate levels categorized by quartile, from regressions adjusted for scaled BMI, square-root transformed and standardized creatinine levels, sex (reference Male), age category (reference 10–19 years), race-ethnicity (reference White) and standardized income-to-poverty ratio, are shown in Fig. 3 and Table 4 Estimates and confidence intervals from the confirmatory multiply imputed study are consistent and are shown in Supplemental Table 4. Results for adjusted quartile-categorized exposure models were significantly stronger than the unadjusted results (Table 3A) and showed the same suggestion of a nonlinear dose‒response, with peak estimates occurring in the third quartile. In the adjusted model, the score estimates increase with age, and related estimates are slightly elevated over reference and over other races for Asian and multiracial participants. This reverses in stratified models. Female sex is protective.

**Table 4:** Associations between standardized metabolic score, glyphosate exposure and covariates (*n* = 5224). The models were adjusted for sex (ref. Male), age category (ref. 10–19 years), race-ethnicity (ref. Non-Hispanic White), scaled BMI, standardized creatinine, and standardized income-poverty ratio

Covariate	Level/Reference	Estimate	95% CI	*p* value
(Intercept)		-0.604	-0.681, -0.527	< 0.0001
Glyphosate Categorized by Quartile Ref: Quartile 1	Quartile 2	0.092	0.029, 0.155	0.0043
	Quartile 3	0.185	0.119, 0.250	< 0.0001
	Quartile 4	0.177	0.107, 0.248	< 0.0001
BMI	(standardized)	0.352	0.328, 0.376	< 0.0001
Creatinine	(sqrt, standardized)	-0.067	-0.094, -0.040	< 0.0001
Sex (Female)	Reference Male	-0.233	-0.278, -0.188	< 0.0001
Age Category Ref: 10–19 years	20–39 years	0.180	0.114, 0.247	< 0.0001
	40–59 years	0.702	0.634, 0.770	< 0.0001
	> 60 years	1.072	1.004, 1.139	< 0.0001
Race/Ethnicity Ref: Non-Hispanic White	Mexican American	0.175	0.108, 0.242	< 0.0001
	Other Hispanic	0.147	0.069, 0.224	0.0002
	Black	0.135	0.074, 0.197	0.0002
	Asian	0.193	0.117, 0.269	< 0.0001
	Multi	0.187	0.081, 0.293	0.00055
Income-Poverty Ratio	(standardized)	-0.049	-0.072, -0.027	0.00002

**Fig. 4:**
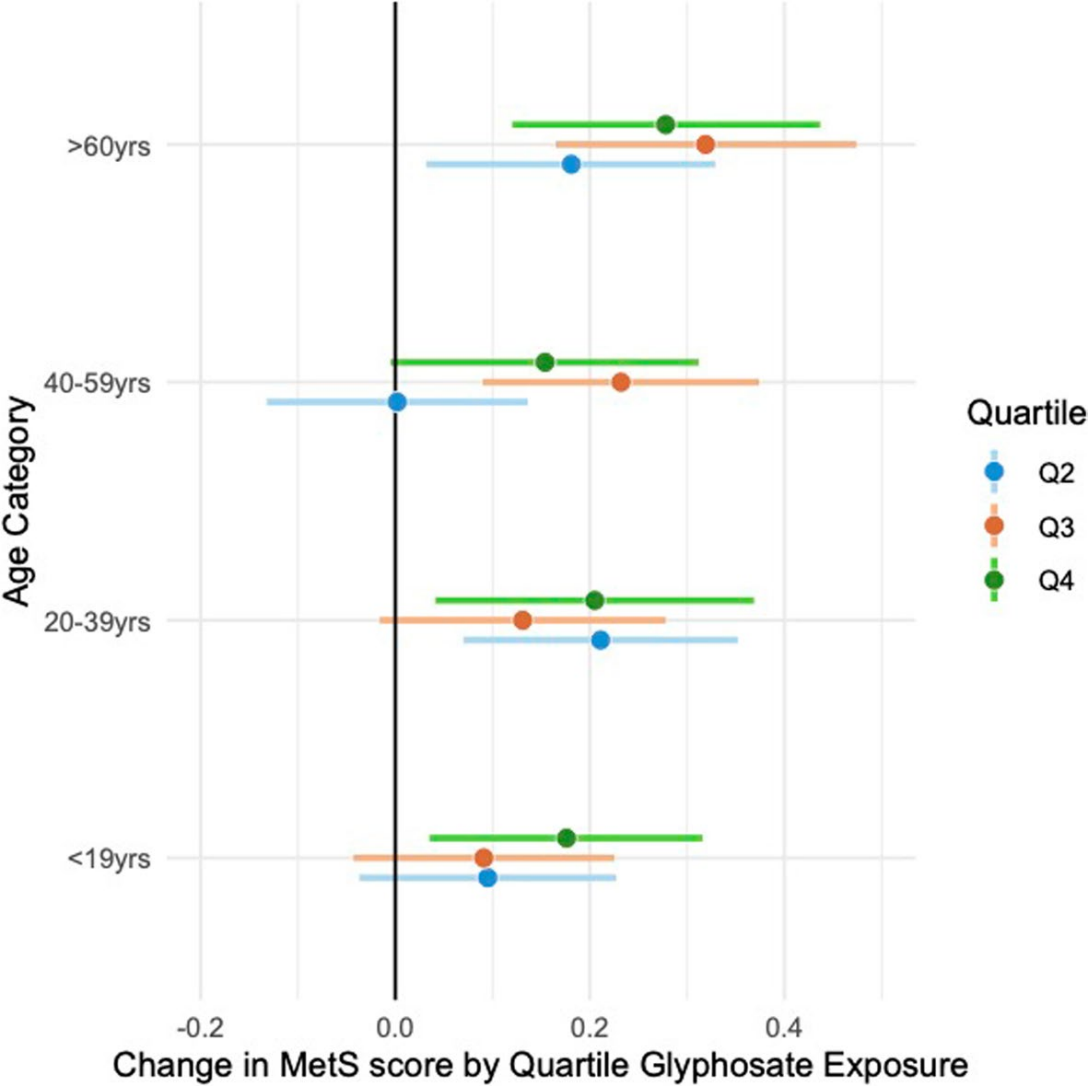
Changes in MetS score by quartile glyphosate exposure from adjusted regression models stratified by age group. The reference level for age in the full model was age 10–19 years (< 19 years). Results from a MetS score derived from multiply imputed risk features are shown in Supplemental Fig. 5A. See supplemental Table 5 for details, including sample sizes and *p* values. To simplify the presentation, only associations for outcome and exposure by quartile are shown for each age grouping

We explored the possibility of effect modification by age, race-ethnicity and sex in a series of stratified, adjusted models. The data were stratified according to the key variable, and models were run separately for each stratum. The results for models stratified by age are shown in Supplemental Table 5. While there are significant differences in quartile estimates by age category (relative to the reference level), confidence intervals overlap. The MetS score increased slightly but inconsistently with age (Fig. 4). Among the 10- to 19-year cohort, only the fourth quartile was significantly different from the reference quartile (0.18, 95% CI = 0.035, 0.316, *p* = 0.015). For the age 20 to 39 cohort, the scores increased significantly in quartile 2, decreased and were marginal in Q3 and increased slightly to become significant in Q4. The cohorts aged 40 years and older had a non-monotonically increasing dose‒response profile. The results are strongest (*p* = 0.00005 at Q3) for participants 60 years and older. We do not adjust *p*-values for multiplicity.

**Fig. 5:**
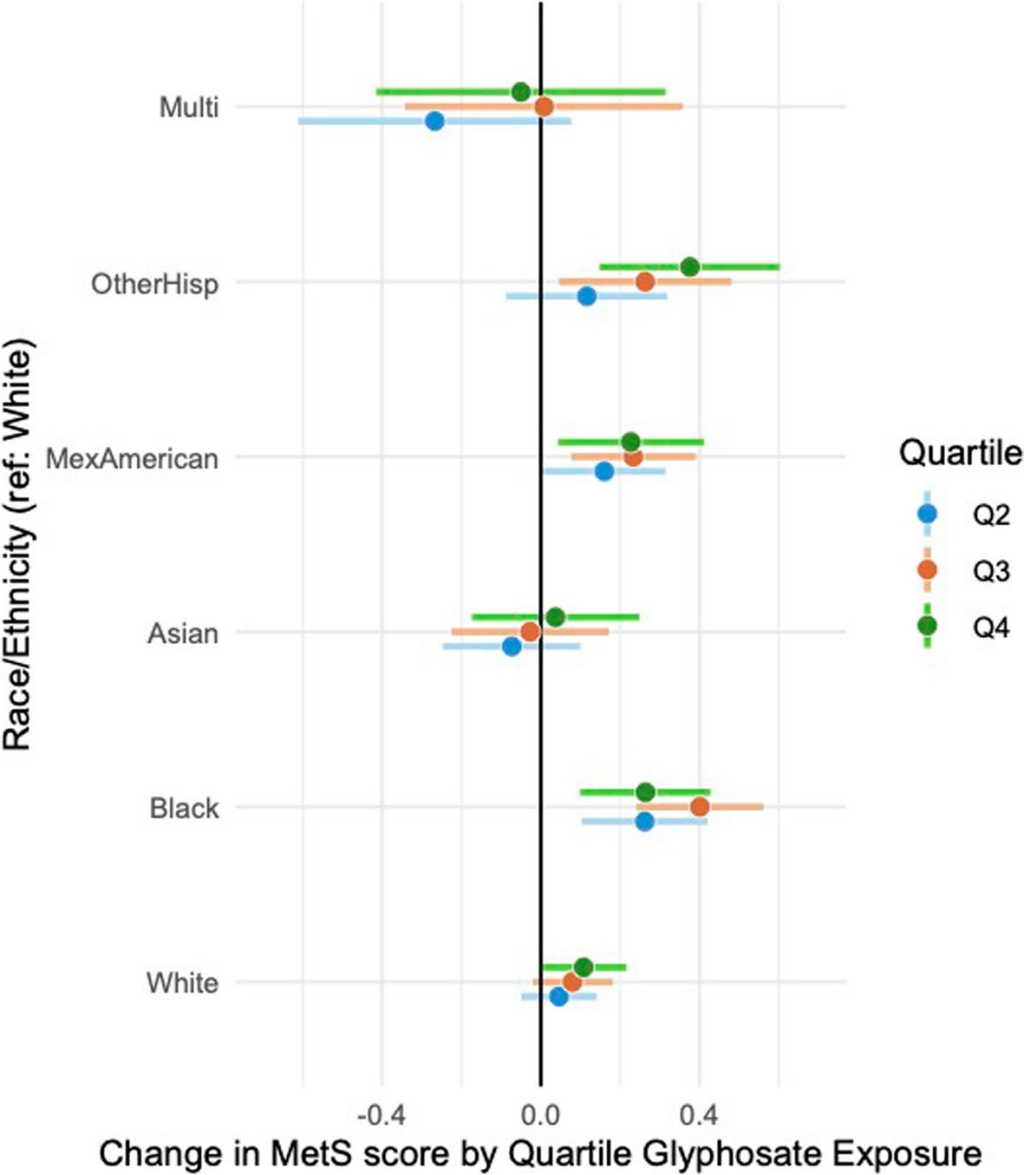
Changes in MetS score by quartile glyphosate exposure from adjusted regression models stratified on race/ethnicity. Results from a MetS score derived from multiply imputed risk features are shown in Supplemental Fig. 5B. See supplemental Table 6 for details including sample sizes and *p* values. To simplify presentation, only associations for outcome and exposure by quartile are shown for each grouping

While confidence intervals for the models stratified on race-ethnicity do overlap, there are two- to threefold differences in the effect sizes, relative to White participants, of estimates for Mexican-Americans (Q4 is 2.1 × greater than reference/White Q4), Other Hispanic (Q4 is 3.5 × larger) and Black participants (Q4 is a factor of 2.44 greater). Significant estimates for Mexican American and Black participants suggest the inverted U-shaped dose‒response profile we observe in the study population as a whole (Fig. 5, Supplemental Table 6).

**Fig. 6:**
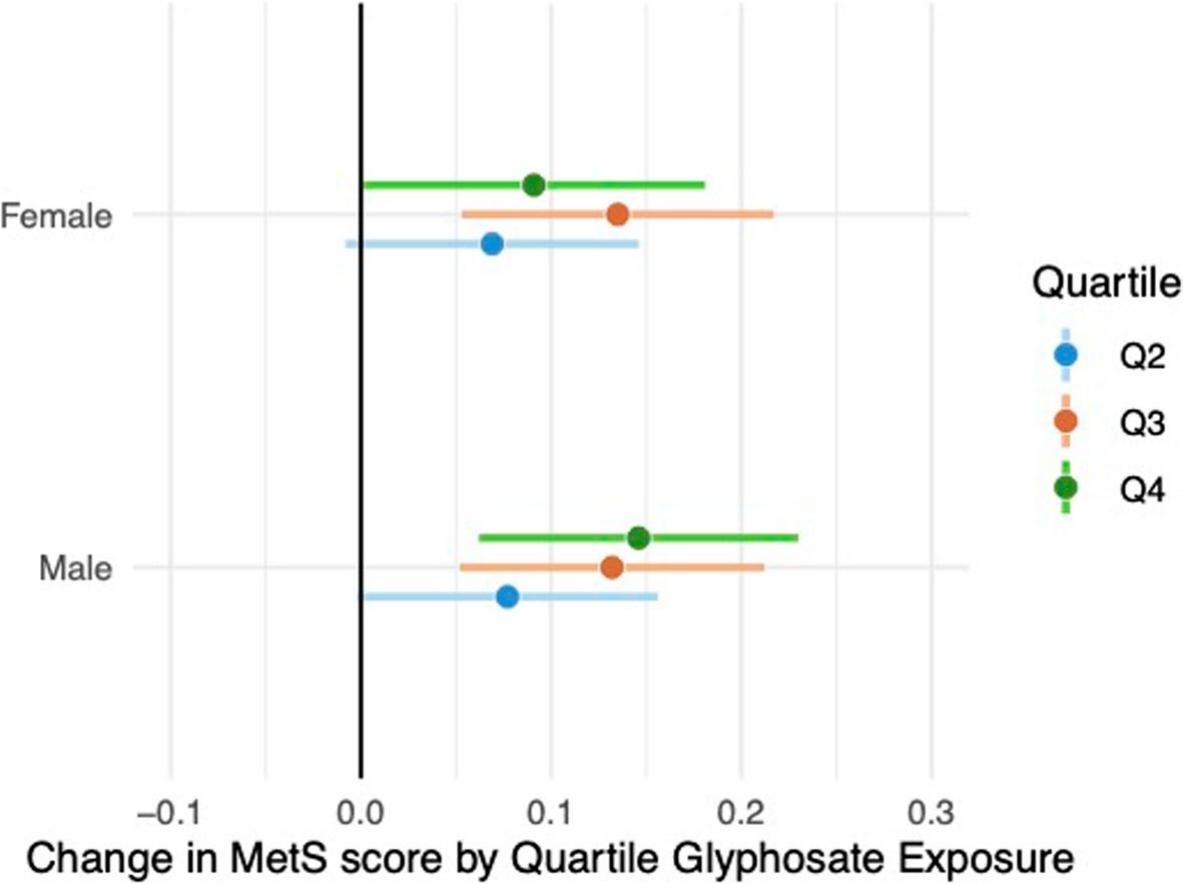
Changes in MetS score by quartile glyphosate exposure from adjusted regression models stratified on sex. Results from a MetS score derived from multiply imputed risk features are d shown in Supplemental Fig. 5C. See supplemental Table 7 for details including sample sizes and *p* values. To simplify presentation, only associations for outcome and exposure by quartile are shown for each grouping

Finally, estimates for models stratified on sex show a significant and inverted U-shaped profile for females and a significant and increasing profile for males (Fig. 6). Aside from the patterns of increase across levels, values are consistent between the sexes (Supplemental Table 7), and confidence intervals overlap, suggesting no evidence of effect modification by sex.

## Discussion

The detailed analysis of associations between urinary glyphosate levels and Metabolic Syndrome (MetS) scores, performed using data from the National Health and Nutrition Examination Survey (NHANES) from 2013 to 2018, revealed significant findings that contribute to the growing body of research on environmental chemical exposure and metabolic health risks. We found significant associations between urinary glyphosate levels and the MetS score, often with a non-monotonic (inverted U-shaped) dose‒response relationship across exposure quartiles. This relationship persisted even after we adjusted for potential confounders, indicating that higher levels of glyphosate exposure are associated with increased MetS risk, peaking at the third quartile of exposure. The observed dose‒response relationship aligns with the notion that moderate levels of exposure may have a more pronounced effect on metabolic health than either low or very high exposures; a nonmonotonic dose‒response relationship (NMDR) suggests a threshold or a range of exposure within which the deleterious effects of glyphosate on metabolic health are most pronounced.

NMDR patterns have been observed in the context of endocrine disruption [60]. Indeed, glyphosate and glyphosate-based herbicides are linked to endocrine disruption in animal models, altering the mRNA and protein expression levels of insulin receptor (IR) and several other receptors and signaling molecules involved in glucose metabolism, such as glucose transporter-2 (GLUT2), JNK, IKKβ, NFkB, IL-6, IL-1β, and TNF-α, as well as transcription factors such as SREBP1c and PPAR-γ [47]. Glyphosate and glyphosate-based herbicides have also been linked to inflammation and cirrhosis of the liver, which leads to the development of insulin resistance and type 2 diabetes in animal models [18, 25, 47]. Our findings suggest the need for further investigation into the mechanistic underpinnings of these relationships and their implications for human health, especially considering the widespread use of glyphosate and the prevalence of metabolic syndrome [32]. A deeper inquiry could yield additional insight into the health effects of glyphosate as well as potential interaction effects with other variables not fully captured in the current study.

Bivariate analysis shows a surprising result for age category, in that the highest exposures occurred among participants aged 10 to 19 years and participants older than 65 years, consistent with exposure results from the 2013–14 NHANES cycle obtained by Ospina et al. [44]. Yet only the older cohort shows very elevated associations between MetS and glyphosate score, perhaps because MetS is not defined among younger individuals [30], and possibly also due to the complex interaction between metabolic syndrome, obesity and aging [5]. Stratified analysis suggested potential effect modification by age and race/ethnicity but not by sex, and the strongest exposure–outcome associations were observed in older participants (60 years and above). These findings align with the broader literature on aging and metabolic health, where older age groups are generally at greater risk for MetS due to various physiological and lifestyle factors [5, 22, 41]. Note that our study omits the age group with the highest levels of documented exposure (aged ~ 10 years or less), with exposure routes through dietary sources such as sweetened ready-to-eat cereal or possibly environmental exposure at school or on recreational grounds (see [44], Table 3).

Significant differences in effect sizes according to race and ethnicity, with notably greater effect sizes for associations between glyphosate exposure categorized by quartile and the MetS score in Mexican-Americans, Other Hispanics, and Black participants, point to potential disparities in susceptibility or exposure to glyphosate. This finding is particularly important given the literature on racial and ethnic disparities in environmental exposures and health outcomes. For instance, Nguyen et al. [43] reported disparities in exposure to various environmental pollutants, including pesticides and herbicides, among racial and ethnic minorities. The present study extends this work by specifically linking these disparities to differential associations with MetS, suggesting that social determinants of health and environmental justice issues are crucial considerations in environmental health research.

The observation that the dose‒response profiles for glyphosate exposure and MetS differ by sex, with a significant and inverted U-shaped profile for females and a linear increase for males, contributes to the growing body of literature on sex-specific health impacts of environmental exposures. This finding aligns with studies highlighting biological and lifestyle differences between sexes that modulate health risks, but it also underscores the need for further research to elucidate the mechanisms underlying these differences, especially in the context of metabolic health and exposure to pollutants.

Unlike many previous studies that have relied on categorical definitions of MetS based on dichotomized risk features, we employ a continuous MetS score derived from EFA from metrics reliably and reproducibly obtained from a large general population. This approach captures nuanced variations in metabolic risk factors, offering a more detailed and sensitive descriptor of MetS risk. The score was subsequently used to explore the change in associations between MetS (represented by the score) and glyphosate by quartile increase in exposure. By stratifying the data on key variables such as age, race-ethnicity, and sex, we explored potential effect modification of the association by demographic features, and the results yielded insights into how the association between glyphosate and MetS varies across different demographic groups and by age. This approach allows for the identification of potentially vulnerable populations and underscores the complexity of the exposure–outcome relationship.

While our study offers significant insights, causal inference is precluded by the cross-sectional design of the NHANES. Additional limitations include that the urine glyphosate concentration was measured only once per participant per cycle and that because it does not bioaccumulate [50, 58], the measured concentration may not accurately reflect long-term exposure or account for variations in individual exposure over time. There may be residual confounding factors in our study, including unmeasured and correlated exposure to other toxins responsible for metabolic dysfunction. We validated the MetS score on only one metric, as no other relevant biometric measurements were supplied by the NHANES for these cycles. Finally, we present an unweighted study.

## Conclusions

We found that urinary glyphosate concentrations are significantly associated with a statistical score designed to capture MetS incidence; in particular, we noted the presence of inverted U-shaped dose‒response relationships with the most pronounced estimates in the third exposure quartile. These findings suggest a complex nonlinear interaction between glyphosate exposure and MetS risk. Our study findings underscore the complexity of the relationship between environmental exposures such as glyphosate and metabolic health, influenced by demographic factors such as age, race-ethnicity, and sex. While our results do not constitute direct evidence, they suggest a need for studies focused on dietary and other glyphosate exposure routes to establish causality, explore the mechanisms driving the observed associations, and address the vulnerabilities of specific demographic groups.

### Supplementary Information

Supplementary Material 1.

Supplementary Material 2.

## Data Availability

The datasets are publicly available from the National Health and Nutrition Examination Survey (NHANES) via the Centers for Disease Control and Prevention (CDC) website: https://www.cdc.gov/nchs/nhanes/Default.aspx.

## Abbreviations

ACR: Urine albumin-to-creatinine ratio
BMI: Body mass index
DBP: Diastolic Blood Pressure
EFA: Exploratory Factor Analysis
GLUT2: Glucose transporter 2
HbA1c: Hemoglobin A1C
HDL: High-density lipoprotein (cholesterol)
IARC: International Agency for Research on Cancer
IDF: International Diabetes Federation
IKKβ: Inhibitory kappa-B kinase beta (regulatory kinase in inflammation)
IL-1β: Interleukin-1β, inflammatory cytokine
IL-6: Interleukin-6, inflammatory cytokine
IR: Insulin Receptor
JNK: A stress-activated protein kinase
KMO: Kaiser–Meyer–Olkin (Measure of Sampling Adequacy test)
LLOD: Lower Limit of Detection
MAP: Mean arterial pressure
MetS: Metabolic syndrome
NHANES: National Health and Nutrition Examination Survey
NFkB: Nuclear factor kappa b (protein transcription factor)
NMDR: Nonmonotonic dose‒response
SBP: Systolic Blood Pressure
SREBP1c: Transcriptional Factor
TNF-α: Tumor necrosis factor-α
