# Supplementary material for: Associations between urine glyphosate levels and metabolic health risks: insights from a large cross-sectional population-based study
Source: Environ Health. 2024 Jun 27;23:58. doi: 10.1186/s12940-024-01098-8 (PMC11210132; doi:10.1186/s12940-024-01098-8)
Supplement: Supplementary file 1 — Supplementary Material 1. [file 12940_2024_1098_MOESM1_ESM.pdf]

Appendix 1. Common Definitions of Metabolic Syndrome

Organization[1]	Obesity	High TG	Low HDL-C	Blood glucose	High blood pressure	Diagnostic criteria
WHO (1998)[2]	Waist/hip ratio > 0.9 in men or > 0.85 in women or BMI > 30 kg/m^2	TG ≥150 mg/dL	HDLC < 40 mg/dl in men or HDL-C < 50 mg/dl in women	IGT, IFG, or T2D	≥140/90 mmHg	IGT, IFG, T2D, or reduced insulin sensitivity plus any two of the criteria
EGIR (1999)[3]	WC >37 inches in men or > 32 inches in women	TG ≥150 mg/dL	<39 mg/dL in men and women	IFG or IGT	≥140 mmHg systolic and ≥ 90 mmHg diastolic or on treatment for HTN	Three or more criteria, including insulin resistance
NCEP ATPIII (2001)[4]	WC ≥102 cm in men or ≥ 88 cm in women	TG ≥150 mg/dl or on therapy lowering TG	<40 mg/dL in men or < 50 mg/dL in women or therapy increasing HDL-C	≥100 mg/dL (including T2D)	≥130/85 mmHg or on treatment for HTN	Three or more criteria
AACE (2003)[5]	BMI ≥25 kg/m^2	≥150 mg/dL	<40 mg/dL in men or < 50 mg/dL in women	IGT or lFG	≥130/85 mmHg	IGT or IFG plus any of the criteria
IDF (2005)[6]	Population-specific increased WC cutoffs	≥150 mg/dL or on TG lowering treatment	<40 mg/dL (men) or < 50 mg/dL (women) or on HDL treatment	IFG or on treatment for hyperglycemia or has T2D diagnosis	≥130 mmHg systolic and/or ≥ 85 mmHg diastolic or on treatment for HTN	Three or more criteria, one of which should be central obesity
IDF (2009)[7]	Population- and country-specific WC cutoffs	TG ≥150 mg/dL	<40 mg/dL in men or < 50 mg/dL in women	≥100 mg/dL	≥130/85 mmHg or on treatment for HTN	Three or more criteria
AHA/NHLBI (2009)[7]	Central obesity WC > 40 inches in men or > 35 inches in women	≥150 mg/dL or treated with lipid lowering drug	<40 mg/dL in men or < 50 mg/dL in women or on treatment	IFG or on treated with diabetes drug or T2D diagnosis	≥130 mmHg systolic and/or ≥ 85 mmHg diastolic or on treatment for HTN	Three or more criteria

[1]***AACE***: *American Association of Clinical Endocrinologists;* ***AHA/NHLBI***: *American Heart Association/National Heart, Lung, and Blood Institute;* ***BMI***: *Body Mass Index;* ***EGIR***: *European Group for the Study of Insulin Resistance;* ***HDL-C***: *High-Density Lipoprotein Choles-terol;* ***HTN***: *Hypertension;* ***IDF***: *International Diabetes Federation;* ***IFG***: *Impaired Fasting Glucose;* ***IGT***: *Impaired Glucose Tolerance* ***NCEP ATPIII***: *National Cholesterol Education Program Adult Treatment Panel III;* ***T2D***: *Type 2 Diabetes;* ***TG***: *Triglycerides;* ***WC***: *Waist Circumference;* ***WHO***: *World Health Organization*

[2]*Alberti KG, Zimmet PZ. Definition, diagnosis and classification of diabetes mellitus and its complications. Part 1: diagnosis and classification of diabetes mellitus provisional report of a WHO consultation. Diabet Med. 1998;***15***:539–553. doi: 10.1002/(SICI)1096-9136(199807)15:7<539::AID-DIA668>3.0.CO;2-S.*

[3]*Balkau B, Charles MA. Comment on the provisional report from the WHO consultation. European Group for the Study of Insulin Resistance (EGIR) Diabet Med. 1999;***16***:442–443.*

[4]*Executive Summary of The Third Report of The National Cholesterol Education Program (NCEP) Expert Panel on Detection, Evaluation, And Treatment of High Blood Cholesterol In Adults (Adult Treatment Panel III) JAMA. 2001;***285***:2486–2497. doi: 10.1001/jama.285.19.2486.*

[5]*Einhorn D, Reaven GM, Cobin RH, et al. American College of Endocrinology position statement on the insulin resistance syndrome. Endocr Pract. 2003;***9***:237–252.*

[6]*Alberti KG et al. The metabolic syndrome: a new worldwide definition. Lancet. 2005;* ***366***:*1059–1062. doi: 10.1016/S0140-6736(05)67402-8*

[7]*Alberti KG, Eckel RH, Grundy SM, et al.. International Diabetes Federation Task Force on Epidemiology and Prevention, National Heart, Lung, and Blood Institute, American Heart Association, World Heart Federation, International Atherosclerosis Society, International Association for the Study of Obesity. Harmonizing the metabolic syndrome: a joint interim statement of the International Diabetes Federation Task Force on Epidemiology and Prevention; National Heart, Lung, and Blood Institute; American Heart Association; World Heart Federation; International Atherosclerosis Society; and International Association for the Study of Obesity. Circulation. 2009;***120***:1640–1645. doi: 10.1161/CIRCULATIONAHA.109.192644.*
