# Supplementary material for: Associations between urine glyphosate levels and metabolic health risks: insights from a large cross-sectional population-based study
Source: Environ Health. 2024 Jun 27;23:58. doi: 10.1186/s12940-024-01098-8 (PMC11210132; doi:10.1186/s12940-024-01098-8)
Supplement: Supplementary file 2 — Supplementary Material 2. [file 12940_2024_1098_MOESM2_ESM.pdf]

Otaru et al. 2024
Supplemental Information

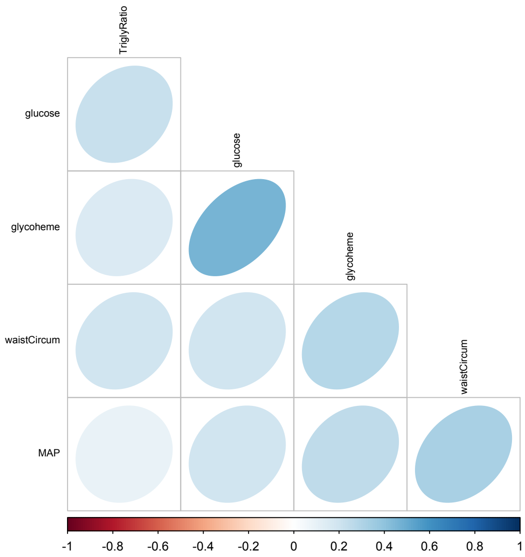

Supplemental Figure 1. Correlogram showing correlations between features used in EFA procedure to create a single score for metabolic syndrome. Ellipses become narrower and darker for higher correlations. Here correlations range from 0.1 to 0.46.

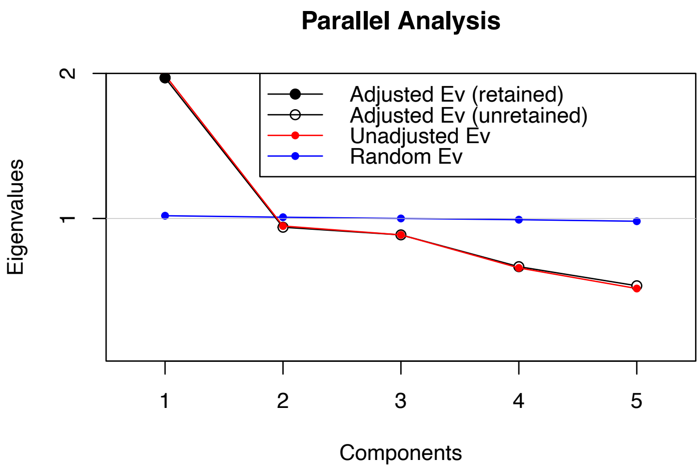

Supplemental Figure 2. Parallel analysis diagram from Horn's Parallel Analysis (Horn, 1965), indicating that one component is sufficient.

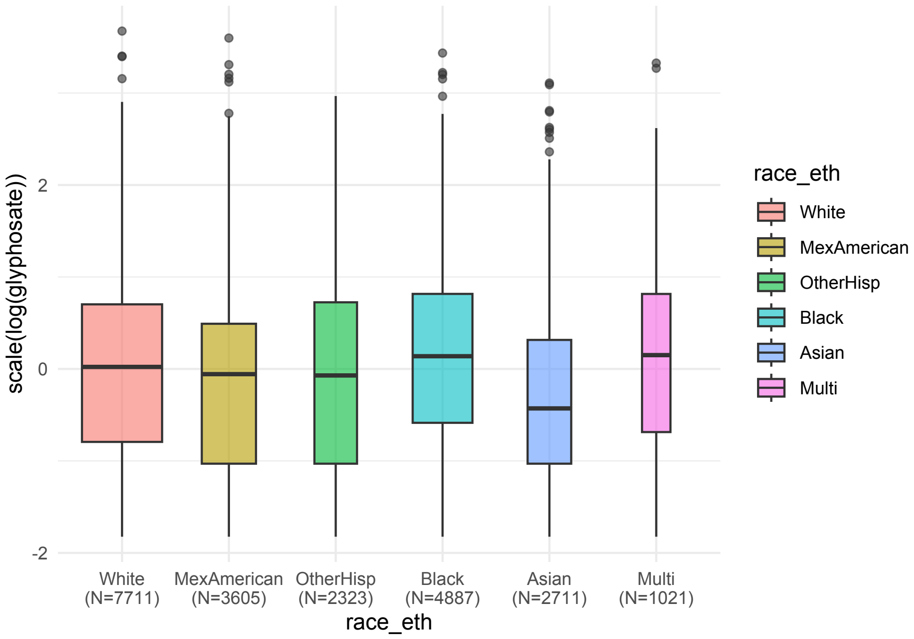

Figure 3A. Log-transformed exposure levels by race**.** Width of each box indicates the relative number of participants per race.

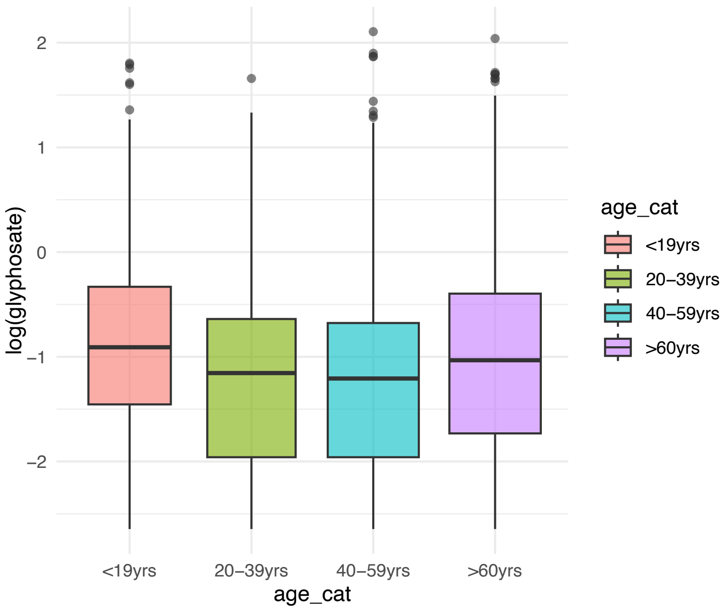

Figure 3B. Log-transformed exposure levels by age category.

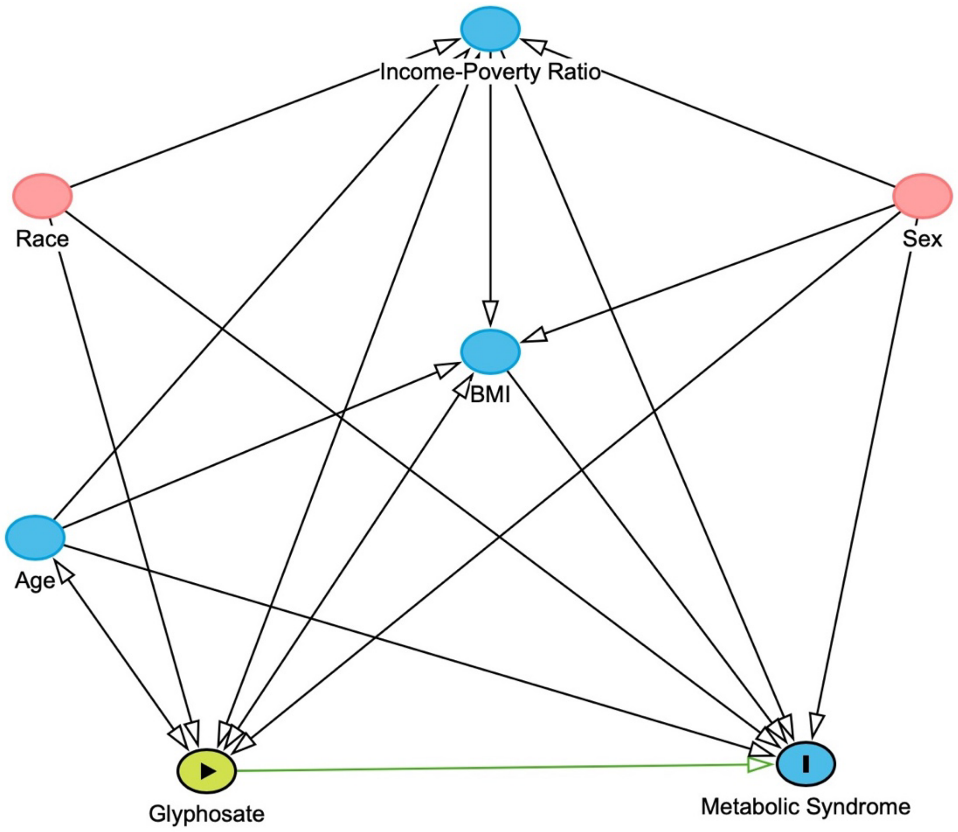

Figure 4.
Directed Acyclic Graph (DAG) showing causal paths for the Glyphosate-MetS system. A sufficient adjusting set for estimating the total effect of glyphosate on Metabolic syndrome includes Age, Race, Sex, Income-Poverty ratio and BMI. BMI may be a mediator but it is not clear to what degree since the interactions between Race, Sex, Age, BMI (obesity) and MetS are complex.

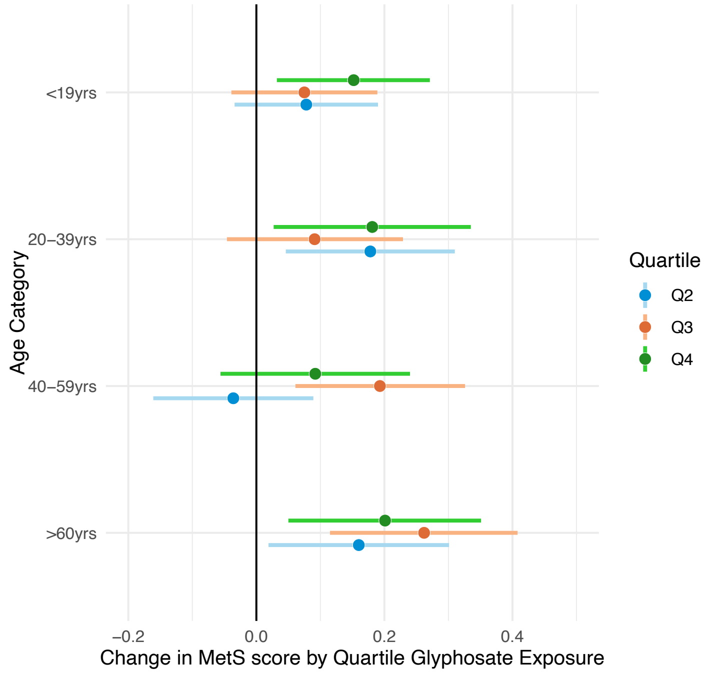

5A. Changes in MetS score by quartile glyphosate exposure from adjusted regression models stratified by age group. MetS score from multiply imputed MetS risk features.

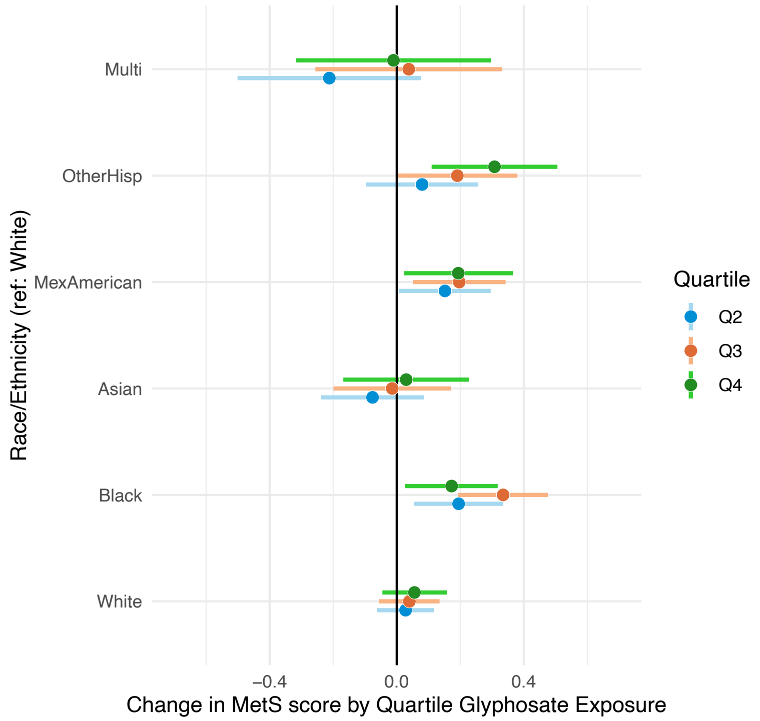

5B. Changes in MetS score by quartile glyphosate exposure from adjusted regression models stratified by race-ethnicity group. MetS score from multiply imputed MetS risk features.

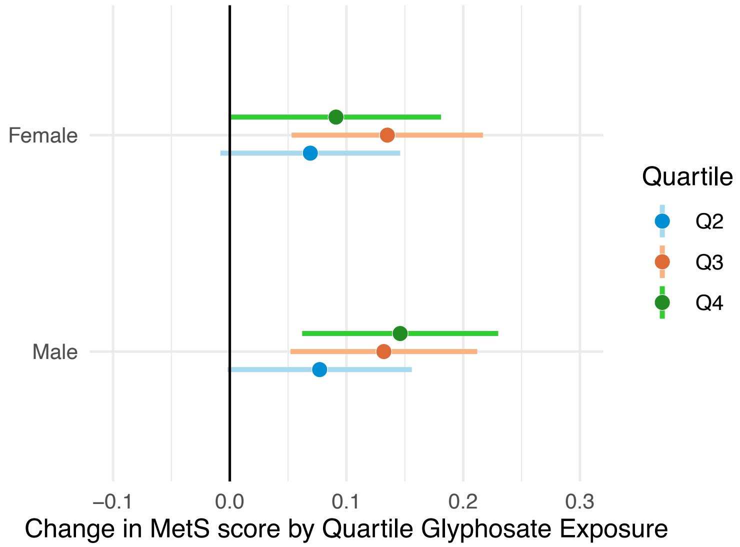

5C. Changes in MetS score by quartile glyphosate exposure from adjusted regression models stratified by sex. MetS score from multiply imputed MetS risk features.

Figure 5A-C.
Changes in MetS score by quartile glyphosate exposure from adjusted regression models stratified by (A) age group, (B), race-ethnicity, and (C) Sex, MetS score from multiply imputed risk features. To simplify the presentation, only associations for outcome and exposure by quartile are shown for each age grouping. See Supplemental Table 4 for full model results from imputed score.

Supplemental Table 1.
Baseline characteristics of study population with missing units summarized.
NHANES cycles 2013 – 2014, 2015-2016, 2017-2018.

**Covariate**	**Level**	**Male** (n = 10797)	**Female** (n = 11461)	**Missing** (%)
Sex	Male	10797 (100.0)	-	-
	Female	-	11461 (100.0)	
Age Category Ref < 19 years	<19yrs	2590 (24.0)	2611 (22.8)	-
	20-39yrs	2687 (24.9)	2907 (25.4)	
	40-59yrs	2612 (24.2)	2959 (25.8)	
	>60yrs	2908 (26.9)	2984 (26.0)	
Race-Ethnicity Ref: NH White	White (ref)	3834 (35.5)	3877 (33.8)	-
	Mexican American	1711 (15.8)	1894 (16.5)	
	Other Hispanic	1049 (9.7)	1274 (11.1)	
	Black	2367 (21.9)	2520 (22.0)	
	Asian	1308 (12.1)	1403 (12.2)	
	Multiracial	528 (4.9)	493 (4.3)	
BMI	Mean (SD)	27.5 (6.76)	28.6 (8.04)	1227 (5.5%)
Income-Poverty Ratio	Med [IQR]	2.0 [1.1, 3.9]	1.88 [1.0, 3.7]	2380 (10.7)
Urine Creatinine		1.31 [0.80 ,1.91]	0.97 [0.53, 1.55]	1493 (6.7)
Urine Glyphosate		0.36 [0.1, 0.65]	0.32 [0.14, 0.57]	16469 (74)
Urine ACR		6.42 [4.3, 12.3]	8.8 [5.7, 16.7]	1493 (6.7)

Supplemental Table 2
Covariates used to create Metabolic Syndrome (MetS) score, summarized by quartile *prior to imputation* and EFA (N=22258). Variables and composite variables used in score are shown boldface. MAP = SBP + 1/3 (SBP - DBP).

Feature	Min	25%	50%	75%	Max	Missing (%)
**Waist circumference**	49.5	81.7	94.4	107	177.9	2156 (9.7%)
Systolic blood pressure	64.67	108	117.33	130	231.33	3254 (14.6)
Diastolic blood pressure	0	60	68	75.33	135.33	3254 (14.6)
**Glycoheme (HbA1c)**	3.5	5.2	5.5	5.8	17.5	3244 (14.6)
**Fasting Glucose**	21	94	100	110	479	13223 (59)
HDL	6	42	51	62	226	2505 (11.3)
Triglycerides	10	58	87	131	4233	13555 (61)
**Triglyceride : HDL Ratio**	0.13	0.99	1.64	2.81	103.24	13555 (61)
**Mean Arterial Pressure (MAP)**	71.56	122.67	134	149.33	279.56	3254 (14.6)

Supplemental Table 3

MetS score validation statistics with performance in classifying MetS-associated renal risk using a logistic model with micro and macroalbuminuria cut-offs as shown (N=20765). Note that not all MetS patients have micro or macroalbuminuria, but likelihood of comorbidity with MetS increases with ACS score (Shi et al. 2020). Diagnostic OR is the ratio of the odds of positivity in those with disease to the odds of positivity in those without (Glas et al. 2003). A value of 1 indicates that the test cannot discriminate, and higher values indicate better discriminatory performance.

ACR cutoff definition	error rate	Sensitivity	Specificity	Accuracy	Diagnostic OR
Microalbuminuria ACR >30 mg/g	12.7%	0.873	0.70	0.873	2.95
Microalbuminuria ACR >100mg/g	4.5%	0.955	0.75	0.955	7.07
Macroalbuminuria ACR > 300 mg/g	2.2%	0.978	0.78	0.978	12.5

Supplemental Table 4

Confirmatory results from a multiple regression model employing a standardized MetS score computed via EFA from 10 multiply imputed (via Hot Deck) datasets are consistent with results from Hot Deck singly imputed data, though Intercept for the score. While some estimates are slightly higher or lower than the single imputed results shown in Table 4, there is no significant difference between results from the multiply imputed score and the single imputed score.

Variable/Covariate	level	Estimate	95% CI	p-value
Glyphosate[1]	Intercept	-0.537	-0.607, -0.467	< 0.0001
	Quartile 2	0.068	0.010, 0.125	0.021
	Quartile 3	0.142	0.082, 0.202	< 0.0001
	Quartile 4	0.126	0.062, 0.190	0.00012
Sex (Ref: Male)	Female	-0.295	-0.336, -0.254	< 0.0001
Age category Ref: 10-19 years	20-39yrs	0.246	0.185, 0.306	< 0.0001
	40-59yrs	0.747	0.686, 0.809	< 0.0001
	>60yrs	1.090	1.029, 1.151	< 0.0001
Race – Ethnicity Ref: Non-Hispanic White	Mexican-American	0.118	0.057, 0.178	0.00015
	Other Hispanic	0.094	0.023, 0.164	0.009
	NH Black	0.059	0.003, 0.114	0.04
	NH Asian	0.134	0.065, 0.203	0.00015
	Multiracial	0.136	0.039, 0.232	0.006
BMI[1]		0.431	0.409 , 0.452	< 0.0001
Creatinine[2]		-0.054	-0.078, -0.029	< 0.0001
Income-poverty ratio[1]		-0.053	-0.073, -0.032	< 0.0001

[1]Standardized

[2]log-transformed, standardized

Supplemental Table 5
Associations between standardized metabolic score and glyphosate, categorized by quartile, adjusted models stratified by age category; score from Hot-Deck single imputed MetS risk metrics. Models are adjusted for standardized BMI, standardized square-root transformed creatinine, sex (reference Male), race-ethnicity (reference Non-Hispanic White), and standardized income-poverty ratio. Since confidence intervals overlap, there is little evidence for effect modification by age, except perhaps for the oldest cohort, which has the strongest associations. Overlap in confidence intervals can be attributed to much reduced sample sizes after stratification. Results are not adjusted for multiple comparisons.

Age Category	**Glyphosate Level**	**Estimate**	**95% CI**	**p-value**
10 to 19 years (n = 1156)	(Intercept)	-0.040	-0.172, 0.091	0.55
	Quartile 2	0.095	-0.037, 0.227	0.16
	Quartile 3	0.091	-0.043, 0.225	0.18
	Quartile 4	**0.176**	**0.035, 0.316**	**0.015**
20 to 39 years (n = 1324)	(Intercept)	-0.002	-0.134, 0.131	0.98
	Quartile 2	**0.211**	**0.070, 0.352**	**0.003**
	Quartile 3	**0.131**	**-0.016, 0.278**	***0.080***
	Quartile 4	**0.205**	**0.041, 0.369**	**0.014**
40 to 59 years (n = 1375)	(Intercept)	-0.088	-0.213, 0.037	0.17
	Quartile 2	0.002	-0.132, 0.136	0.97
	Quartile 3	**0.232**	**0.090, 0.374**	**0.0014**
	Quartile 4	**0.154**	**-0.005, 0.312**	***0.05***
60 years and above (n = 1369)	(Intercept)	-0.203	-0.342, -0.063	**0.0044**
	Quartile 2	**0.181**	**0.032, 0.329**	**0.017**
	Quartile 3	**0.319**	**0.165, 0.474**	**0.00005**
	Quartile 4	**0.278**	**0.120, 0.437**	**0.0006**

Supplemental Table 6

Associations between standardized metabolic score and glyphosate, categorized by quartile, adjusted models stratified by race-ethnicity categories. Models are adjusted by standardized BMI, standardize creatinine, sex (reference Male), age category (reference 10 to 19 years) and standardized income-poverty ratio.

Race-Ethnicity	Level	Estimate	95% CI	p-value
White (n = 1979)	(Intercept)	-0.434	-0.545, -0.322	<0.00001
	Quartile 2	0.046	-0.05, 0.141	0.35
	Quartile 3	0.080	-0.02, 0.181	0.12
	Quartile 4	**0.108**	**0.00, 0.216**	**0.049**
Mexican American (n = 833)	(Intercept)	-0.528	-0.683, -0.373	<0.00001
	Quartile 2	**0.160**	**0.004, 0.315**	***0.045***
	Quartile 3	**0.233**	**0.077, 0.390**	**0.0035**
	Quartile 4	**0.227**	**0.043, 0.412**	**0.016**
Other Hispanic (n = 523)	(Intercept)	-0.582	-0.813 -0.350	<0.00001
	Quartile 2	0.116	-0.087, 0.319	0.26
	Quartile 3	**0.263**	**0.046, 0.481**	**0.018**
	Quartile 4	**0.376**	**0.148, 0.603**	**0.0014**
Black (n = 1071)	(Intercept)	-0.657	-0.832, -0.482	<0.00001
	Quartile 2	**0.262**	**0.103, 0.421**	**0.0012**
	Quartile 3	**0.401**	**0.241, 0.561**	**<0.00001**
	Quartile 4	**0.264**	**0.099, 0.428**	**0.0017**
Asian (n = 569)	(Intercept)	-0.364	-0.564, -0.164	0.0004
	Quartile 2	-0.073	-0.247, 0.100	0.41
	Quartile 3	-0.027	-0.225, 0.171	0.79
	Quartile 4	0.037	-0.174, 0.249	0.73
Multiracial (n = 249)	(Intercept)	-0.141	-0.472, 0.190	0.40
	Quartile 2	-0.267	-0.611, 0.077	0.13
	Quartile 3	0.008	-0.342, 0.358	0.96
	Quartile 4	-0.050	-0.415, 0.315	0.79

Supplemental Table 7

Associations between standardized metabolic score and glyphosate, categorized by quartile, adjusted models stratified by sex. While there are differences by sex, confidence intervals overlap, so there is little evidence of effect modification by sex. Models are adjusted by standardized BMI, standardized square-root transformed creatinine, sex (reference Male), age category (reference 10-19 years), race-ethnicity (reference Non-Hispanic White), and standardized income-poverty ratio.

Sex	Level	Estimate	95% CI	p-value
Female (n = 2619)	(Intercept)	-0.675	-0.785, -0.564	<0.00001
	Quartile 2	0.092	0.003, 0.182	0.043
	Quartile 3	0.193	0.098, 0.288	0.00074
	Quartile 4	0.157	0.052, 0.262	0.0033
Male (n = 2605)	(Intercept)	-0.77	-0.873, -0.668	<0.00001
	Quartile 2	0.097	0.007, 0.187	*0.035*
	Quartile 3	0.176	0.084, 0.267	0.00016
	Quartile 4	0.192	0.097, 0.288	0.00008
